# Coronavirus nucleocapsid proteins assemble constitutively in high molecular oligomers

**DOI:** 10.1038/s41598-017-06062-w

**Published:** 2017-07-18

**Authors:** Yingying Cong, Franziska Kriegenburg, Cornelis A. M. de Haan, Fulvio Reggiori

**Affiliations:** 1 Department of Cell Biology, University Medical Center Groningen, University of Groningen, A. Deusinglaan 1, 9713 AV Groningen, The Netherlands; 2 Virology Division, Department of Infectious Diseases & Immunology, Faculty of Veterinary Medicine, Utrecht University, Utrecht, The Netherlands

## Abstract

Coronaviruses (CoV) are enveloped viruses and rely on their nucleocapsid N protein to incorporate the positive-stranded genomic RNA into the virions. CoV N proteins form oligomers but the mechanism and relevance underlying their multimerization remain to be fully understood. Using *in vitro* pull-down experiments and density glycerol gradients, we found that at least 3 regions distributed over its entire length mediate the self-interaction of mouse hepatitis virus (MHV) and severe acute respiratory syndrome coronavirus (SARS-CoV) N protein. The fact that these regions can bind reciprocally between themselves provides a possible molecular basis for N protein oligomerization. Interestingly, cytoplasmic N molecules of MHV-infected cells constitutively assemble into oligomers through a process that does not require binding to genomic RNA. Based on our data, we propose a model where constitutive N protein oligomerization allows the optimal loading of the genomic viral RNA into a ribonucleoprotein complex via the presentation of multiple viral RNA binding motifs.

## Introduction

*Coronaviruses* (CoV) are enveloped positive-stranded RNA viruses and *Coronaviridae* can be subdivided into four groups based on phylogenetic clustering: *alpha*-, *beta*-, *gamma*- and *delta*- CoV^1, 2^. Members of this virus family infect the mammalian respiratory and gastrointestinal tracts by incompletely understood mechanisms^2, 3^. The relevance of this virus family has considerably increased due to the recent emergence of the severe acute respiratory syndrome (SARS) and Middle East respiratory syndrome (MERS), which are caused by viruses belonging to the *beta*-CoV group^4, 5^. The Mouse Hepatitis Virus (MHV) is closely related to SARS-CoV and MERS-CoV, and considered the prototype for the investigation of the CoV life cycle^6^.

The proteins encoded by the CoV genomic RNA (gRNA) can be divided into two major categories. The first entails the 15 or 16 nonstructural proteins (nsp1 to nsp15/16)^1, 2, 7^, which are synthesized in the host cell and assemble into the replicase-transcriptase complexes (RTCs). RTCs are associated with and/or are embedded into double-membrane vesicles (DMVs) and convoluted membranes, which are generated during CoV infection and very likely act as replication platforms^8^. The second category contains the structural and accessory proteins. A minimal set of 4 structural proteins is critically required for the efficient formation of infectious virions^1, 2, 7^. Those include the envelope (E), the membrane (M), the spike (S) and the nucleocapsid (N) proteins.

**Figure 1:**
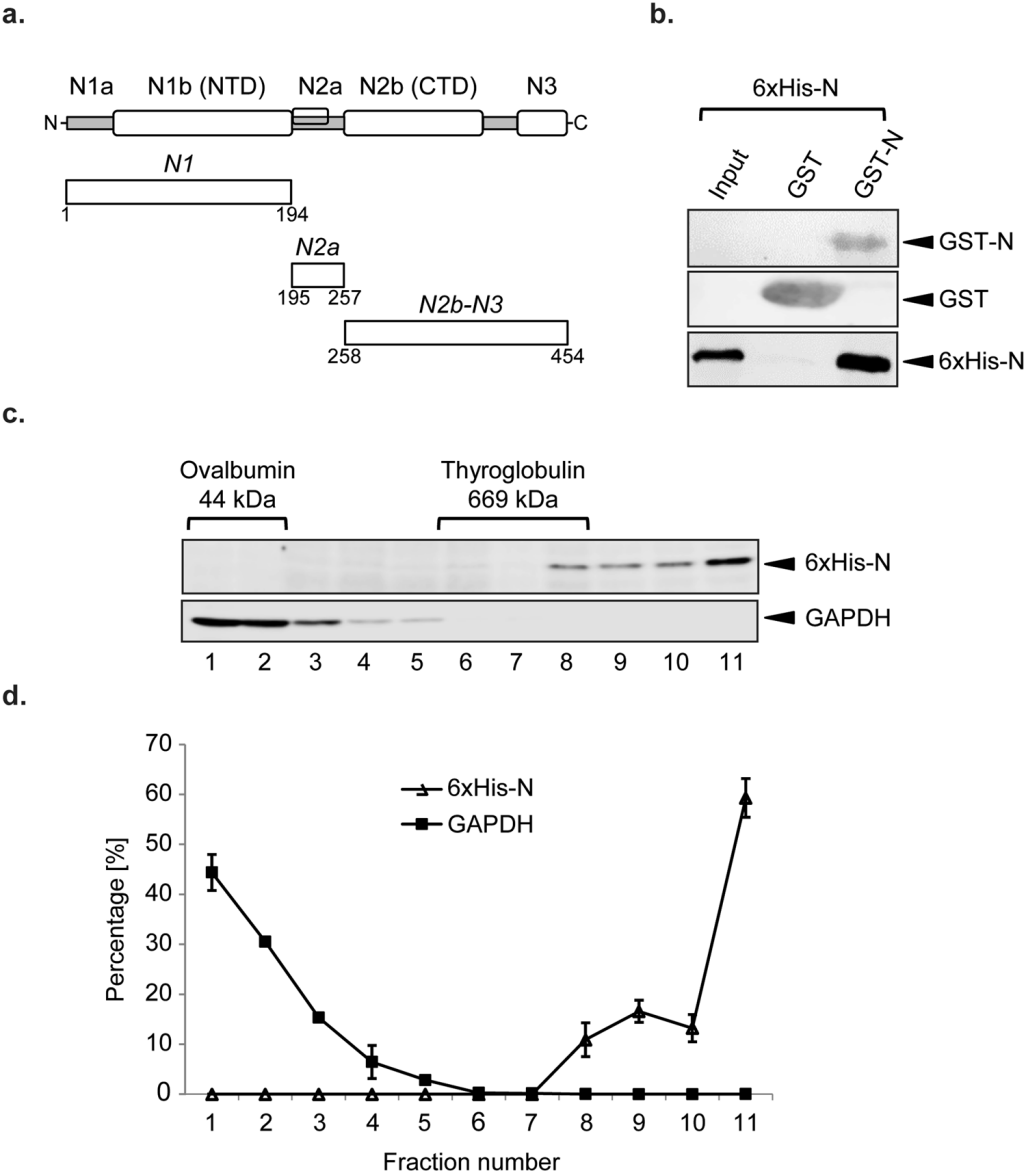
Recombinant MHV N protein forms large oligomers. (**a**) Schematic structural organization of the MHV N protein and overview of the truncations generated in this study (modified from ref. 9). (**b**) Bacterial extract from *E*. *coli* expressing 6xHis-tagged MHV N protein (input) was incubated with immobilized GST or GST-N protein. Precipitated proteins were eluted in SDS-sample buffer and analyzed by western blot using an anti-His monoclonal antibody. Only part of the western blot images is shown in the figure. The GST and GST-N amount were assessed by staining the PVDF membrane with Ponceau Red. (**c**) Bacterial extract from *E*. *coli* expressing 6xHis-tagged MHV N protein was sedimented in a 5–20% glycerol gradient at 50,000 g for 75 min. Eleven fractions were collected and protein content analysed using antibodies against the 6xHis tag. Gradient and centrifugation conditions were assessed by sedimenting a LR7 cell extract and probing the collected fractions with a GAPDH antibody. Only part of the western blot images is shown in the figure. (**d**) Quantification of the immunoblots presented in panel (c) plus standard deviation (SD) (n = 3).

The N protein is the only structural protein that associates with RTCs^9–11^. It binds the gRNA and it is essential for the incorporation of the virus genetic material into CoV particles^12, 13^. Moreover, it is the major component of ribonucleoprotein complex sitting in the virion cores^2, 7, 14^ and thus also plays an essential architectural role in the virus particle structural organization through a network of interactions with the gRNA, the M protein and other N molecules^7, 8, 15^. The MHV N protein has been divided into multiple domains based on genetic analyses and structural studies^16–20^. The two largest domains, the N-terminal domain (NTD), also N1b, and the C-terminal domain (CTD), also N2b, fold independently and have gRNA-binding properties^16–18, 21^ (Fig. 1a). These two regions are flanked by the N-terminal N1a domain, the centrally located N2a domain, and the C-terminal B spacer and N3 domain^21^ (Fig. 1a).

It has been hypothesized that dimerization and possibly oligomerization of CoV N proteins plays an essential role in virus particle assembly^21–25^. The ability of SARS-CoV N protein to self-interact was first demonstrated using yeast two-hybrid and co-immunoprecipitation experiments^26^. The crystal structures of the N2b/CTD domain of SARS-CoV and MHV N proteins confirmed this observation, leading to the notion that N protein dimers are the basic building blocks of the ribonucleoprotein complex^12, 23, 24, 27–29^. Subsequently, native gel electrophoresis, size exclusion chromatography and surface plasmon resonance revealed that recombinant SARS-CoV N protein forms oligomers *in vitro*, although it appears to predominantly exist as a dimer in solution in absence of gRNA^30^. Size exclusion chromatography and chemical cross-linking assays were also used to unveil that the N2b/CTD domain of the SARS-CoV N protein, in particular the stretch of amino acids between positions 343–402, forms autonomously oligomers in solution^31, 32^. The N2b/CTD domain of the MHV N protein has also been shown to bind full length protein^11^. However, few other studies indicated that other parts of the N protein could also be involved in the self-interaction. In particular, the N1b/NTD and N3 domains could bind full length MHV N protein^11, 19^ while the serine-rich (SR) region located within the N2a could be essential for SAR-CoV N protein self-interaction and oligomerization^33^. Nonetheless, the current most commonly accepted working model is that CoV N protein constitutively dimerizes, primarily via the N2b/CTD domain, and subsequently oligomerizes during virion assembly through a mechanism that remains unclear^22, 23, 27, 29, 32, 34–36^. It is also unclear whether gRNA binding influences CoV N protein oligomerization.

In this study, we confirm that recombinant MHV and SARS-CoV N protein self-interact to form large oligomers. However, we could also show that, besides N2b/CTD, several domains of the N protein are involved in this process. Analysis of different MHV and SARS-CoV N protein truncations revealed that at least three regions of these proteins cross-interact between each other in an interchangeable manner. Moreover, we show that two of these regions, i.e. N1 and N2b-N3, can oligomerize autonomously. Further, in infected cells, the MHV N protein forms oligomers already in the cytoplasm and its oligomerization does not require binding to gRNA. Altogether these findings indicate that CoV N proteins self-interact and oligomerize via discontinuous regions present in domains distributed over the entire protein to generate large supra-complexes. We hypothesize that these oligomers, which are formed constitutively, provide a larger binding surface for the gRNA, which will be thus optimally engaged at the RTCs and subsequently incorporated into forming viral particles.

## Results

### MHV N protein forms large oligomers *in vitro*

To gain insights into the self-assembly mechanism of the N protein, we decided to use an *in vitro* approach to exclude the eventual involvement of other factors in this event. Since order-disorder and secondary structure predictions coupled with sequence alignment has highlighted that all CoV N proteins have the modular organization as MHV N protein, we used this as a model first. Hence, MHV N protein was expressed in *E*. *coli* as a GST fusion construct and purified using glutathione Sepharose (GSH-beads). The immobilized GST fusion protein or GST was incubated with bacterial cell extract of *E*. *coli* expressing recombinant 6xHis-tagged full length MHV N protein. As illustrated in Fig. 1b, recombinant 6xHis-tagged N protein specifically bound GST-N protein but not GST alone, which is in agreement with previous reports^19, 23, 24, 27–29^ and further shows that N proteins self-interact directly.

Since it has been shown that CoV N proteins also bind non-viral RNA^19, 21, 37–40^, we also investigated the association between purified GST-tagged and 6xHis-tagged N proteins after RNase A treatment to exclude a role of bacterial RNA in the assayed interaction. As shown in Supplementary Fig. S1a, RNase A treatment removed large part of the RNA present in the bacterial extract and nucleic acids were not detected associated to the purified N protein even when this was not exposed to RNase A. Importantly, removal of RNA did not affect the binding between the two N protein fusions (Supplementary Fig. S1b). We concluded that the bacterial RNA does not participate in the *in vitro* interaction of MHV N protein.

To determine whether the N-protein form homo-dimers or homo-oligomers, we sedimented recombinant 6xHis-tagged N protein on a continuous 5–20% glycerol gradient For comparison sedimentation of the protein standards ovalbumin (44 kDa) and thyroglobulin (669 kDa) was analysed, which could be recovered in fractions 1–2 and 6–8, respectively (Fig. 1c and d). GAPDH, which is present in the cytoplasm as a monomer^41^, was detected in the first fractions of a sedimentation performed with a LR7 cell extract. In contrast, the 6xHis-tagged N protein was exclusively found in fractions 8–11, indicating that it forms large homo-oligomers (Fig. 1c and d).

### MHV N protein assembles in cytoplasmic oligomers over the course of an infection

**Figure 2:**
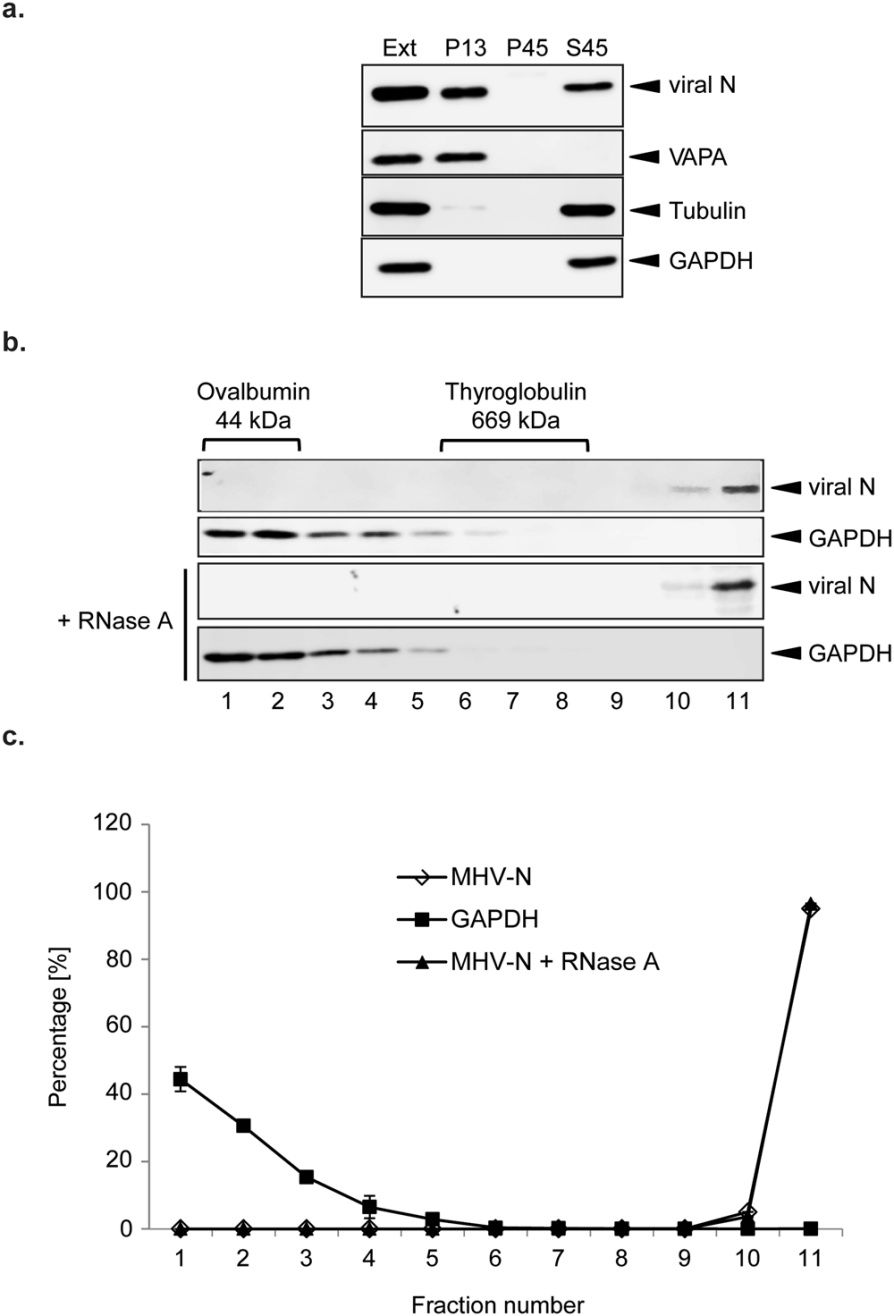
MHV N protein assembles in cytoplasmic oligomers over the course of an infection. (**a**) Cleared lysate from MHV-infected LR7 cells (Ext) was centrifuged at 15,000 × *g* for 10 min to obtain a pellet (P13) and a supernatant, which was subsequently centrifuged at 110,000 × *g* for 1 h to be separated into a pellet (P45) and a supernatant (S45). Equivalent amounts of each fraction were separated by SDS-PAGE and analysed by western blot using antibodies against MHV N protein, tubulin (cytoplasm), GAPDH (cytoplasm) and VAPA (endoplasmic reticulum). Only part of the western blot images is shown in the figure. (**b**) The S45 fraction obtained in panel (a) was mocked treated or treated with RNase A for 30 min on ice before sedimentation and analysed as in Fig. 1c. A260/280 ratios <0.1 was used to assess complete hydrolysis of the nucleic acids. Only part of the western blot images is shown in the figure. (**c**) Quantification of the immunoblots presented in panel (b) plus SD (n = 3).

We explored whether the N protein also forms oligomers over the course of an infection. In MHV-infected cells, the N protein localizes to three distinct locations: RTCs, virions and cytoplasm^2, 9, 10, 13, 42^. Since the N protein is in large complexes with other viral proteins in both RTCs and virions, we decided to focus on its cytoplasmic pool and to separate it from the two other populations through differential centrifugations. We generated cell extracts (Ext) from MHV-infected LR7 cells and centrifuged them at 15,000 × *g* to discard the pellet P13, which includes compartments with low sedimentation rates such as whole cells or debris, nuclei and mitochondria. The resulting supernatant S13 was subsequently centrifuged at 110,000 × *g* to sediment organelles such as the Golgi apparatus but also virions that could have remained associated to the cell surface at the moment of the lysis^43, 44^. Successful fractionation of each step was followed by visualization of the membrane bound protein VAPA for P13^45^ as well as the cytoplasmic proteins GAPDH and tubulin for S45, which also contained soluble N protein (Fig. 2a). Thus, we concluded that the cytoplasmic pool of the N protein is enriched in the S45 supernatant.

The S45 supernatant was then applied onto the same continuous 5–20% glycerol gradient employed for the analysis of the size of recombinant N protein complexes. Interestingly, cytoplasmic N protein from MHV-infected cells was exclusively detected in the last fractions of the gradient similarly to recombinant N protein, while GAPDH was found only in the low-density fractions (Fig. 2b and c). A small difference in size between the MHV N protein oligomers formed *in vitro* and *in vivo*, however, was detected. This could be due to either a slight inhibition of recombinant N protein self-interaction caused by the 6xHis tag, or a better oligomerization *in vivo* because of cellular factors such as molecular chaperones. Moreover, it cannot be excluded that there are host proteins that associate to N protein oligomers.

The S45 supernatant was also incubated with RNase A to degrade all nucleic acids (Supplementary Fig. S1c), before applying the sample onto the glycerol gradient to examine whether N protein oligomerization is influenced by binding to gRNA *in vivo*. This treatment, however, did not change the sedimentation profile of the N protein (Fig. 2b and c). Altogether these data show that the MHV N protein forms large cytoplasmic oligomers in infected cells, and that this aggregation does not depend on its binding to gRNA. Moreover, this result underlines the validity of using recombinant N protein to study its oligomerization determinants.

### Multiple domains mediate N protein oligomerization

**Figure 3:**
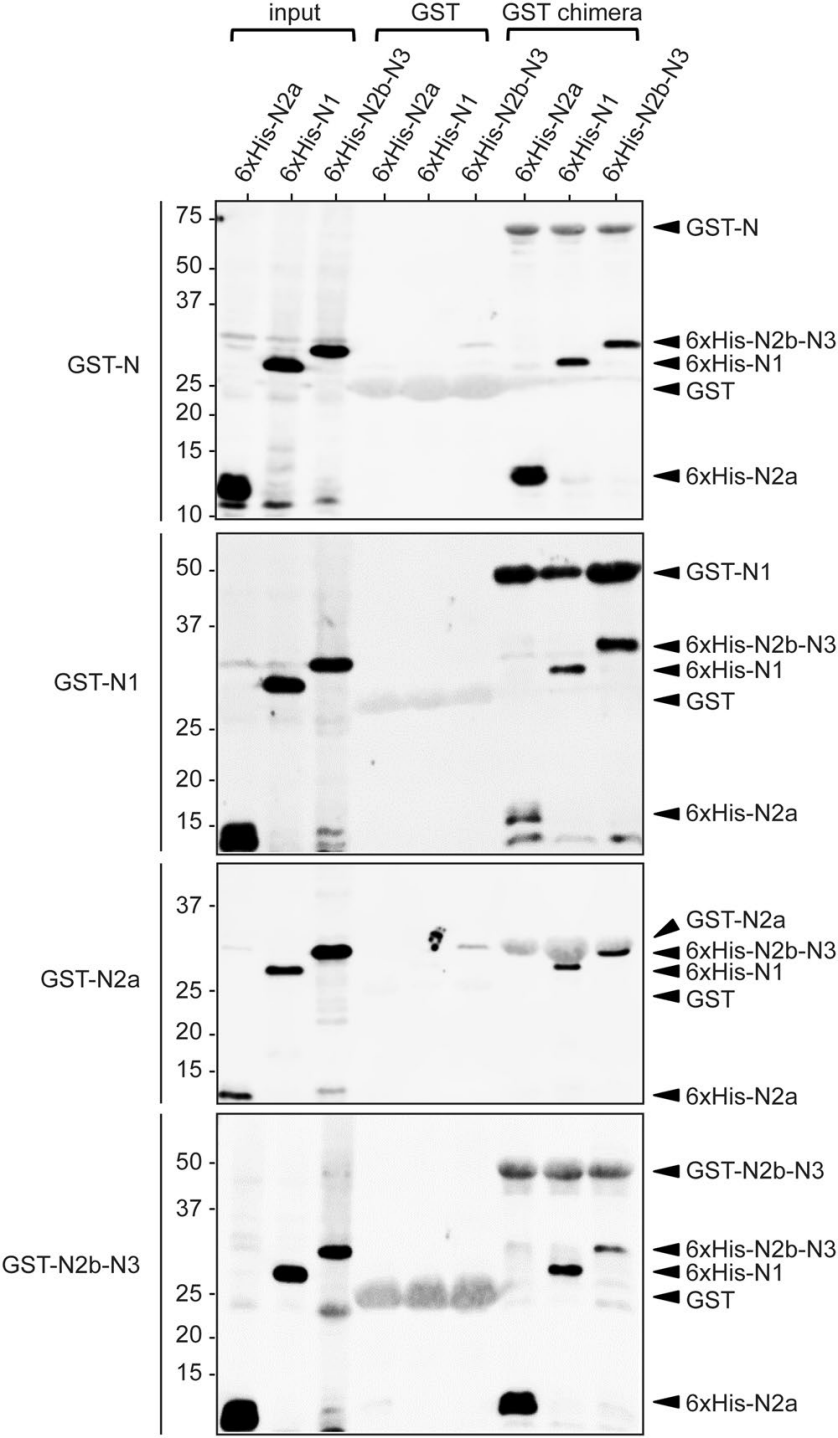
Multiple domains mediate N protein oligomerization. Bacterial extracts from *E*. *coli* expressing the 6xHis-tagged N1, N2a and N2b-N3 truncations were incubated with immobilized GST, GST-N protein, GST-N1, GST-N2a and GST-N2b-N3. Precipitated proteins were eluted in SDS-sample buffer and analyzed by western blot using the anti-6xHis monoclonal antibody. A260/280 ratios <0.1 indicated the absence of nucleic acids in the samples.

Previous studies have shown that the N2b/CTD domain is required for the dimerization of N proteins of different CoVs^12, 23, 24, 27, 28^. Our consistent finding that the N protein forms oligomers suggested that there might be several domains involved in the self-interaction. We thus generated three 6xHis-tagged truncations, i.e. N1 (which contains the NTD), N2a and N2b-N3 (which contains the CTD), which collectively cover the full length of the N protein (Fig. 1a). These constructs were expressed in *E*. *coli* and the resulting bacterial extracts were incubated with either immobilized GST or GST-N protein. Interestingly, all three analyzed N protein truncations specifically bound GST-N protein but not GST alone (Fig. 3, top panel), further revealing that in addition to the reported N2b/CTD region, the N protein possesses binding domains for the self-interaction in the N1 and N2a parts as well.

Next we explored whether there was any putative redundancy in binding between the different domains of the N protein to mediate N protein oligomerization. For this, bacterial cell extracts from *E*. *coli* expressing 6xHis-tagged N1, N2a or N2b-N3 were incubated with immobilized GST or GST-N protein as well as the truncations GST-N1, GST-N2a and GST-N2b-N3. Interestingly, all constructs were able to specifically bind both the N1 and N2b-N3 truncations suggesting that each domain within the N protein could interact with at least two different regions from another N protein molecule (Fig. 3). Further, 6xHis-tagged N1 and 6xHis-tagged N2b-N3 displayed a binding with immobilized GST-N2a. However, there was no binding between 6xHis-tagged N2a and immobilized GST-N2a indicating that the N2a domain might participate to the oligomer formation without being one of the critical determinants. 6xHis-tagged N2b-N3 was pulled down by GST-tagged N1 protein irrespectively of RNase A treatment confirming that the studied bindings do not depend on bacterial RNA (Supplementary Fig. S1d).

To determine whether the analyzed truncations also form oligomers, we sedimented bacterial extract from *E*. *coli* expressing 6xHis-tagged N1, N2a or N2b-N3 truncations on a continuous 5–20% glycerol gradient. As shown in Supplementary Fig. S2a,b, the 6xHis-tagged N1 and 6xHis-tagged N2b-N3 truncations are both forming oligomers. In contrast, however the 6xHis-tagged N2a sediments at lower molecular weight fractions. The presence of the 6xHis-tagged N2a fusion protein in fraction 3–4 also suggest that the N2a fragment might be able to multimerize through probably a weak interaction that could not be detected by pull-down experiments. From these findings we concluded that MHV N protein oligomerizes via multiple discontinuous regions.

### SARS-CoV N protein is also forming oligomers *in vitro*

**Figure 4:**
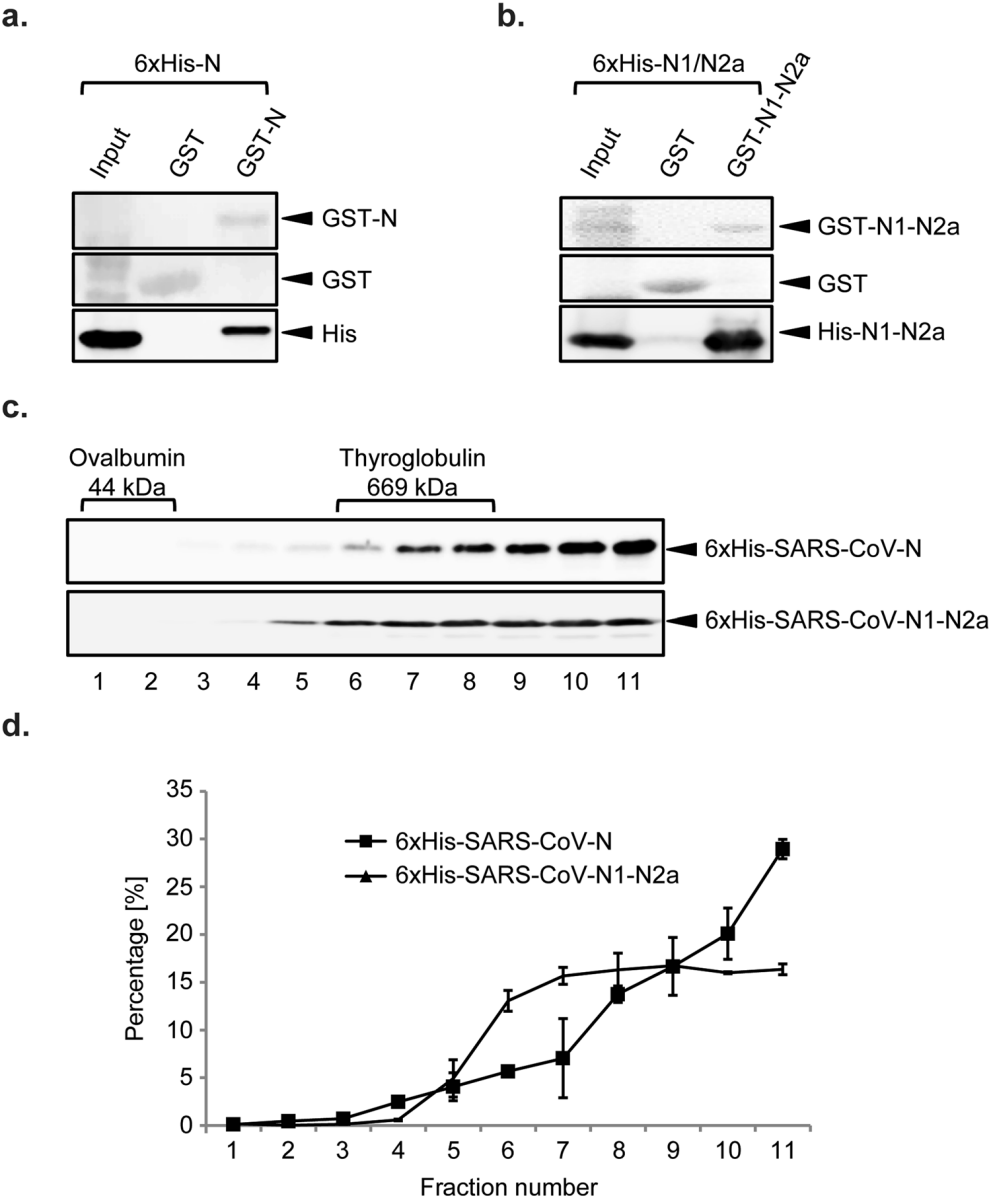
SARS-CoV N protein is also forming oligomers. (**a**) Bacterial extract from *E*. *coli* expressing 6xHis-tagged SARS-CoV N protein (input) was incubated with immobilized GST or GST-SARS-CoV-N protein. Precipitated proteins were eluted in SDS-sample buffer and analyzed by western blot using an anti-His monoclonal antibody. The amounts of GST and GST-N were assessed by staining the PVDF membrane with Ponceau Red. A260/280 ratios <0.1 indicated the absence of nucleic acids in the samples. Only part of the western blot images is shown in the figure. (**b**) Bacterial extracts from *E*. *coli* expressing 6xHis-tagged SARS-CoV N1-N2a were processed and analyzed as in panel (a). A260/280 ratios <0.1 indicated absence of nucleic acids in the samples. Only part of the western blot images is shown in the figure. (**c**) Bacterial extracts of *E*. *coli* expressing 6xHis-tagged SARS N protein and N1-N2a truncation were sedimented in a 5–20% glycerol gradient at 50,000 g for 75 min. Eleven fractions were collected and protein content analyzed using antibodies against the 6xHis tag. Gradient and centrifugation conditions were assessed by sedimenting a LR7 cell extract and probing the collected fractions with a GAPDH antibody. Only part of the western blot images is shown in the figure. (**d**) Quantification of the immunoblots presented in panel (c) plus standard deviation (SD) (n = 3).

To determine whether other CoV N proteins have the same characteristic, we analyzed the SARS-CoV N protein. First, recombinant 6xHis-tagged SARS-CoV N protein was incubated with immobilized GST or GST-tagged SARS-CoV N protein. As shown in Fig. 4a, recombinant SARS-CoV N protein specifically bound to GST-SARS-CoV N protein but not GST, confirming that SARS-CoV N protein self-interacts^12, 23, 24, 27, 28^. Subsequently, bacterial extract from *E*. *coli* expressing 6xHis-tagged SARS-CoV N protein was applied onto a 5–20% glycerol gradient. The 6xHis-tagged SARS-CoV N protein was mainly detected in the late fractions of the gradient (Fig. 4c and d). These results showed that the SARS N protein, similarly to the MHV N protein, forms high molecular weight oligomers. Those of SARS-CoV N protein, however, appear to be smaller and this could be due to either the difference in size between SARS-CoV and MHV N proteins (423 amino acids versus 455) or the fact that SARS-CoV N protein forms smaller oligomers.

Previous data have shown that the N2b/CTD domain of SARS-CoV N protein domain self-interacts^24, 26, 31, 34^. To explore whether the N-terminus of the SARS-CoV N protein also participates in its oligomerization, as seen for the MHV N protein, we performed pull-down experiments with recombinant 6xHis-tagged SARS-CoV-N1-N2a and immobilized GST-SARS-CoV-N1-N2a. As depicted in Fig. 4b, recombinant SARS-CoV N1-N2a protein specifically binds to GST-SARS-CoV N1-N2a protein but not GST alone, revealing that the N-terminal N1-N2a domain self-interacts. Moreover, analysis of recombinant 6xHis-tagged SARS-CoV-N1-N2a on glycerol gradients showed that this truncation forms high molecular weight oligomers (Fig. 4d and e). Similarly to MHV, however, SARS-CoV-N2a was unable to self-interact and form oligomers (Supplementary Fig. S3). Altogether our results confirm that the SARS-CoV N protein oligomerizes but the oligomerization process involves not only the CTD domain but also the N-terminal area of the N protein, which contains the NTD domain.

## Discussion

Using pull-down experiments and density glycerol gradients, our study provides additional evidence that CoV N proteins oligomerize. Several structural biology studies have indicated that SARS-CoV N protein dimerizes through the N2b/CTD domain^22, 24, 27, 32, 36^ and it is generally accepted that the dimerization of N2b/CTD domain serves as the basic building block for CoV ribonucleoprotein virion core formation through multimerization^15, 46, 47^. We and others, however, have already pointed out that the N-terminal N1b/NTD and N3 regions could also interact with full length of MHV N protein^11, 19^. Our data extends and completes the information acquired by these investigations as we found that at least three areas of MHV N protein, i.e. N1a-N1b, N2a and N2b-N3, mediate its self-interaction. Importantly, those domains can bind reciprocally between themselves (Fig. 3) and our findings provide a possible model for MHV N protein oligomerization, i.e. a single N molecule appears to have several binding sites that allow the association of multiple N protein units to a single oligomer. However, we cannot exclude that binding promiscuousity and affinity of the various N protein domains, which we have investigated using truncated proteins, are more limited and different, respectively, in the context of the full-length protein.

Although N1a-N1b and N2b-N3 regions are able to oligomerize independently, N2a is not and therefore this domain probably only contributes to and/or reinforces MHV N protein self-interaction (Fig. 3 and Supplementary Figs S2 and S3). This is in agreement with a report indicating that the N2a domain, in particular the SR region within it, could play a role for SARS-CoV N protein self-interaction and oligomerization^33^. Further, the N2a domain is known to interact with the nonstructural protein nsp3 via a serine and arginine rich stretch within that domain^9–11^. Therefore, one can speculate that N2a is more important for the association of the N protein to the RTCs rather than providing another oligomerization platform.

Our attempts to narrow down the specific binding area within the various regions have been unsuccessful since it appears that the amino acids crucial for the N protein self-interaction are discontinuously distributed (Fig. 3). The results obtained with the N2a truncation indirectly support this notion as a short domain will possess less key binding amino acids and consequently it will interact less pronouncedly compared to longer parts such as the N1 and N2b-N3 domains. Previous efforts to localize the residues essential to mediate SARS-CoV N2b-N3 (including the CTD) multimerization identified three different regions within this domain^22, 31, 34^, further underscoring the notion that discontinues binding regions, distributed over the entire N molecule, are responsible for the self-association. During the preparation of this manuscript, a cryo-EM analysis of ribonucleoprotein complexes isolated MHV virions was published and the model emerging from this work is consistent with our findings^48^. This study suggested that the N protein form octamers mainly via its CTD domain, which then further assembles into larger oligomeric structures that can acquire either a loose or a more compact intertwined filament shape^48^. In the proposed model, multiple surfaces of the N protein participate in the multimerization of the N protein octamer and this is coherent with our conclusion that several domains in the N protein mediate self-interaction.

One important finding of our study is that cytoplasmic MHV N protein forms high molecular weight oligomers in infected cells. Similarly to the recombinant protein, we could not detect monomers, dimers or small multimers on glycerol gradients (Fig. 2b) suggesting that after synthesis, MHV N protein rapidly assembles into oligomers. It is easy to imagine that, based on our *in vitro* data, this is very likely also the case for the SARS-CoV N protein. Since all CoV N proteins have an identical modular organization^21^ and have the N1b, N2a and N2b domains, oligomerization could be a characteristic that all of them possess. It has been suggested that gRNA promotes MHV N protein self-interaction because the association process was partially or largely susceptible to RNase A treatment^11, 35^. Another study, however, reached the opposite conclusion^19^, which is also supported by structural biology studies where N protein multimers have been detected and analyzed in preparations that do not contain RNA^12, 22–24, 27, 29, 32, 36, 49^. Our data showing that both in *vivo* and *in vitro* N protein oligomerization does not depend on its binding to gRNA are in agreement with this latter conclusion. It cannot be excluded, however, that association to gRNA could promote further N protein oligomerization. This could explain the partial discrepancy with the study showing that RNase A treatment interferes with the binding between the N protein and full-length N protein or N1b/NTD domain, but not with the N2b/CTD region^11^.

**Figure 5:**
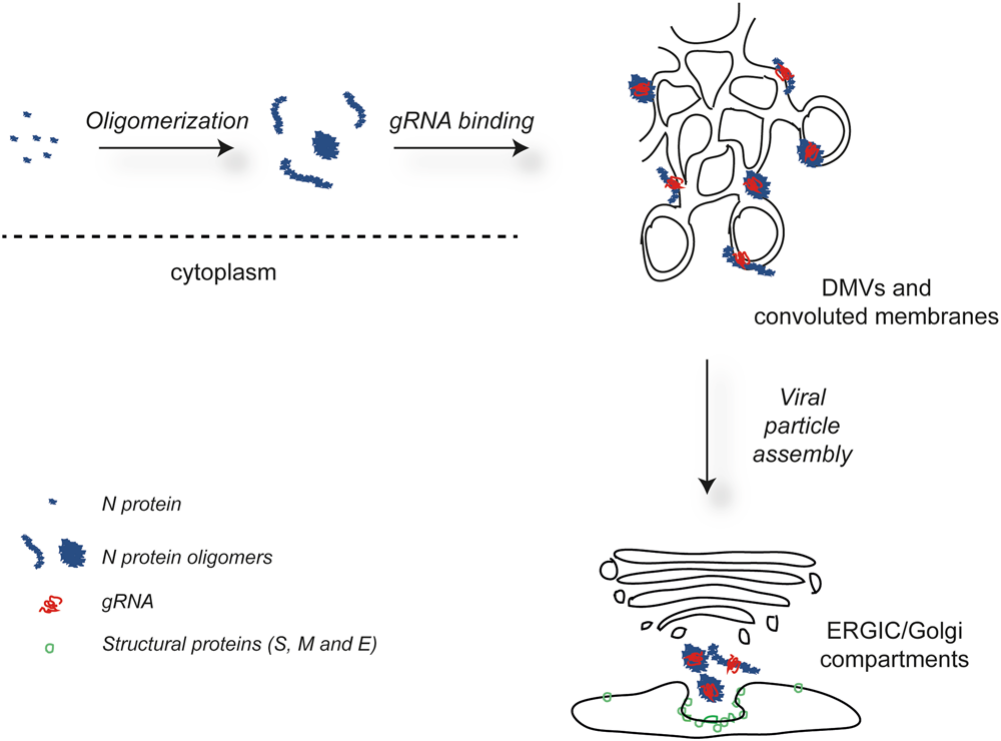
Models for the role of CoV N proteins over the course of an infection. After synthesis, CoV N proteins constitutively assemble into oligomers with loose or more compact intertwined filament shapes^48^, which are recruited to the RTCs localized on double-membrane vesicles (DMVs) and convoluted membranes via their interaction with nsp3. At these replication platforms, newly synthesized gRNA is engaged by N protein oligomers, which co-operate with the rest of the structural proteins to form the viral particles at the ERGIC/Golgi compartments.

Which could be the relevance of constitutive N protein olimerization? RNA chaperones are nonspecific nucleic acid binding proteins with long disordered regions that help RNA molecules to adopt its functional conformation^50–52^. In agreement with this notion, which has already been proposed for CoV N proteins^40, 53^, our hypothesis is that recruitment of already formed N protein oligomers to the RTCs at DMVs and convoluted membranes via the interaction with nsp3^9, 10^, allows efficient and tight loading of the exceptionally large gRNA via numerous binding sites into a ribonucleoprotein complex (Fig. 5). This RNA chaperone role of N protein oligomers would assure the efficient incorporation of the gRNA into the assembling virions, other scenarios, however, are also possible and future investigations will help to decipher the functional relevance of CoV N protein oligomerization.

## Materials and Methods

### Cell culture and virus

LR7 cells were maintained in Dulbecco’s Modified Eagle Medium (DMEM; Cambrex Bioscience, Walkersville, MD) supplemented with 10% fetal calf serum (Bodinco Alkmaar, The Netherlands), 100 IU of penicillin/ml and 100 µg/ml of streptomycin (both from Life Technologies, Rochester, NY). Wild type MHV-A59 was propagated in LR7 cells in DMEM.

### Plasmids

The sequences coding for either full-length MHV N protein or its truncations, i.e. N1 (amino acids 1 to 194), N2a (amino acids 195 to 257) and N2b-N3 (amino acids 258 to 454), were amplified by PCR from MHV gRNA and cloned into pET32c (EMD Millipore, Amsterdam, The Netherlands) and pGEX (GE Healthcare, Little Chalfont, United Kingdom) vectors using *Bam*HI and *Xho*I, creating the pET32c-N, pET32C-N1, pET32C-N2a, pET32C-N2b-N3, pGEX-N, pGEX-N1, pGEX-N2a and pGEX-N2b-N3 constructs. The SARS-CoV N protein coding sequence or its truncations N1-N2a (amino acids 1 to 260) and N2a (amino acids 189 to 260) were also amplified by PCR and cloned into pET32c and pGEX vectors using *Xho*I and *Not*I to create pET32c-SARS-CoV-N, pET32c-SARS-CoV-N1-N2a, pGEX-SARS-CoV-N, pGEX-SARS-CoV-N1-N2a and pGEX-SARS-CoV-N2a.

### Bacterial extracts

Transformed *Escherichia coli* BL-21 were grown in 125 ml of LB medium (0.5% yeast extract, 1% tryptone, 1% NaCl) to late exponential phase and after inducing protein expression by addition of 0.5 mM isopropyl-β-D-thiogalactopyranoside, cells were grown at 37 °C or 20 °C for 4 h or 16 h, respectively. Bacteria were harvested, resuspended in 4 ml lysis buffer (PBS, 5 mM DTT, 1 mg/ml lysozyme, 1 mM PMSF, 10% glycerol, 1% Triton X-100 and complete protease inhibitor (Roche) and lysed by two sonication rounds of 10 sec using a Branson sonicator (Danbury, Connecticut, United States). The bacterial lysates were cleared by centrifugation at 15,000 × *g* for 10 min at 4 °C and passed through a 0.45 µm filter. For purification of GST fusion proteins, lysates were incubated with 125 µl of glutathione (GSH) Sepharose (4B, GE Healthcare), which had been pre-washed in PBS. Where indicated, lysates were incubated with 40 mg/ml RNase A (Invitrogen, Carlsbad, CA) for 30 min on ice prior to addition to the GSH Sepharose. Cell extracts from bacteria expressing the 6xHis-tagged proteins were used either directly for pull-down experiments or for the purification of the fusion proteins with nickel Sepharose (6 Fast Flow, GE Healthcare) after incubation in presence or absence of 40 mg/ml RNase A for 30 min on ice. Complete hydrolysis of RNA was verified by sample analysis on an agarose gel followed by nucleic acid staining with Midori Green (GC Biotech, Netherlands) or determination of the A260/A280 ratio through measurement of A260 and A280 using a NanoDrop Spectrophotometer (Implen, Germany)^54–56^.

### Cell extracts

For the preparation of cell extracts, LR7 cells grown on 10 cm dishes were mocked-treated or inoculated with MHV at a MOI of 1 and after 8 h, they were lysed by 5 min sonication in 1.2 ml of PBS buffer supplemented as described above. Supernatants were then cleared by centrifugation at 15,000 × *g* for 10 min at 4 °C and passed through a 0.45 µm filter. RNase A treatments were carried out by incubating 200 µl of cell extract with 40 mg/ml of enzyme for 30 min on ice, immediately prior to pull-downs.

### Pull-down experiments

For the pull-down experiments, GSH-Sepharose bound GST fusion protein were incubated with 200 µl of bacterial extract or 200 µl of LR7 cell extracts on a rotatory wheel for 2 h at 4 °C, subsequently washed at 4 °C three times in PBS supplemented with 5 mM DTT, 10% glycerol, 1% Triton X-100 and one time in PBS buffer. Proteins bound to the Sepharose beads were eluted in 20 µl of sample buffer by boiling and subjected to SDS-PAGE, blotted onto PVDF membranes and visualized by either membrane staining with Ponceau Red or western blot analysis using anti-6xHis antibody (HIS H8, Thermo, Waltham, MA) or anti-N protein monoclonal antibodies^11^. Bound primary antibodies were detected using the Alexa680-conjugated goat polyclonal anti-mouse IgG antibody (Life Technologies) and signals visualized with an Odyssey system (LI-COR, Lincoln, NE).

### Subcellular fractionation and glycerol gradient sedimentation

Cell extracts (Ext) from MHV-infected LR7 cells were centrifuged at 15,000 × *g* for 10 min (4 °C) to obtain a pellet (P13) and a supernatant (S13), which was further centrifuged at 110,000 × *g* for 60 min (4 °C) to also get a pellet (P45) and a supernatant (S45). Proportional aliquots of Ext, P13, P45 and S45 fractions were examined by resolving them by SDS-PAGE and then by probing western blot membranes with monoclonal antibodies against MHV N protein and polyclonal antisera against GAPDH (Fitzgerald, North Acton, MA), tubulin (Sigma-Aldrich, St. Louis, MO) or VAPA (Santa Cruz, Dallas, TX).

For glycerol gradient sedimentation, 100 µl of either the S45 fraction or bacterial extracts expressing 6xHis-tagged full-length or truncated proteins were loaded on the top of a 2,2 ml continuous 5–20% glycerol gradient in lysis buffer (w/v) prepared using the Gradient Master machine (Biocomp, New Brunswick, Canada). After centrifugation at 135,000 × *g* for 75 min at 4 °C in a TLS55 rotor (Beckman Coulter, Brea, CA), 11 fractions of 200 µl were collected from the top to the bottom of the gradient. After precipitation by addition of 20 µl of tri-chloroacetic acid (final concentration 10%), proteins were resolved by SDS-PAGE and analyzed by western blot using antibodies against the N protein, GAPDH and the 6xHis tag. Thyroglobulin (669 kDa) and ovalbumin (44 kDa) (Bio-Rad, Berkeley, CA) were used as molecular weight protein standards to determine the gradient resolution.

## Electronic supplementary material

Supplementary information
