# Supplementary material for: Coronavirus nucleocapsid proteins assemble constitutively in high molecular oligomers
Source: Sci Rep. 2017 Jul 18;7:5740. doi: 10.1038/s41598-017-06062-w (PMC5515880; doi:10.1038/s41598-017-06062-w)
Supplement: Supplementary file 1 — Supplementary information [file 41598_2017_6062_MOESM1_ESM.pdf]

Supplementary Information

Coronavirus nucleocapsid proteins assemble constitutively in high molecular oligomers

Yingying Cong, Franziska Kriegenburg, Cornelis A.M. de Haan and Fulvio Reggiori

Supplementary Inventory

(1) Supplementary Figures

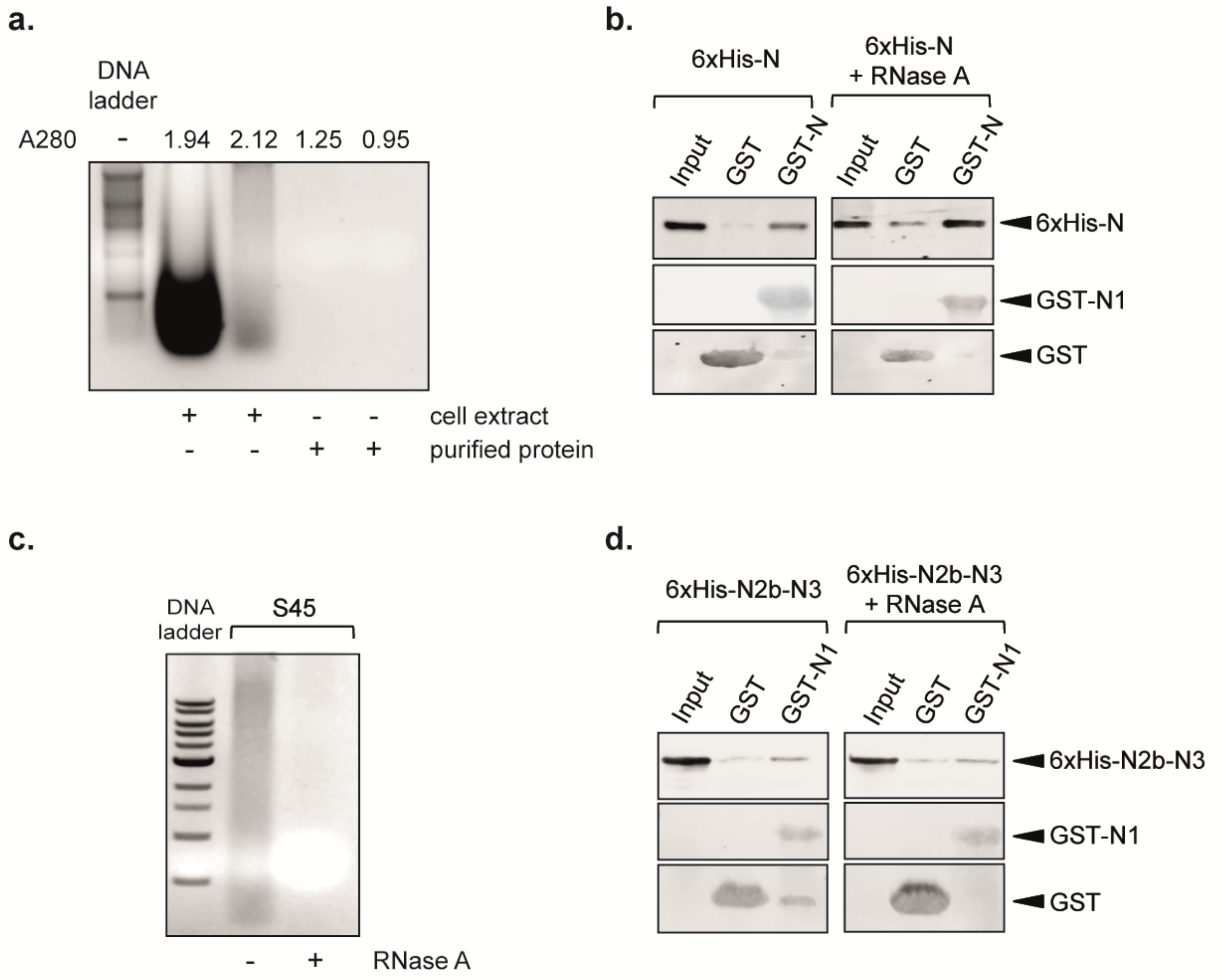

Supplementary Figure S1. ***E. coli*** **RNA does not influence the self-interaction of purified MHV N protein.** (a, b) Bacterial extracts from *E. coli* expressing the 6xHis-tagged N proteins were kept on ice in presence or absence of RNase A for 30 min, before to be purified using nickel Sepharose beads. In parallel, extracts from bacteria expressing GST or GST-N protein were also subjected to RNase A treatment or not prior purification. (a) 10 μl of cell extracts or purified protein were loaded and separated on a 1% agarose gel, which was finally stained for nucleic acids using Midori Green. Equivalent amounts of loaded protein were estimated by measuring the A280. (b) Purified 6xHis-tagged N protein was incubated with immobilized GST and GST-N proteins. Bound proteins were eluted in SDS-sample buffer and analyzed by western blot using the anti-6xHis monoclonal antibody. Only part of the western blot images are shown in the figure. (c) Purified 6xHis-tagged N2b-N3 protein was incubated with

immobilized GST and GST-N1 proteins. These preparations were treated or not with RNase A. Bound proteins were eluted in SDS-sample buffer and analyzed by western blot using the anti-6xHis monoclonal antibody. A260/280 ratios < 0.1 indicated absence of nucleic acids in the samples. Only part of the western blot images are shown in the figure.

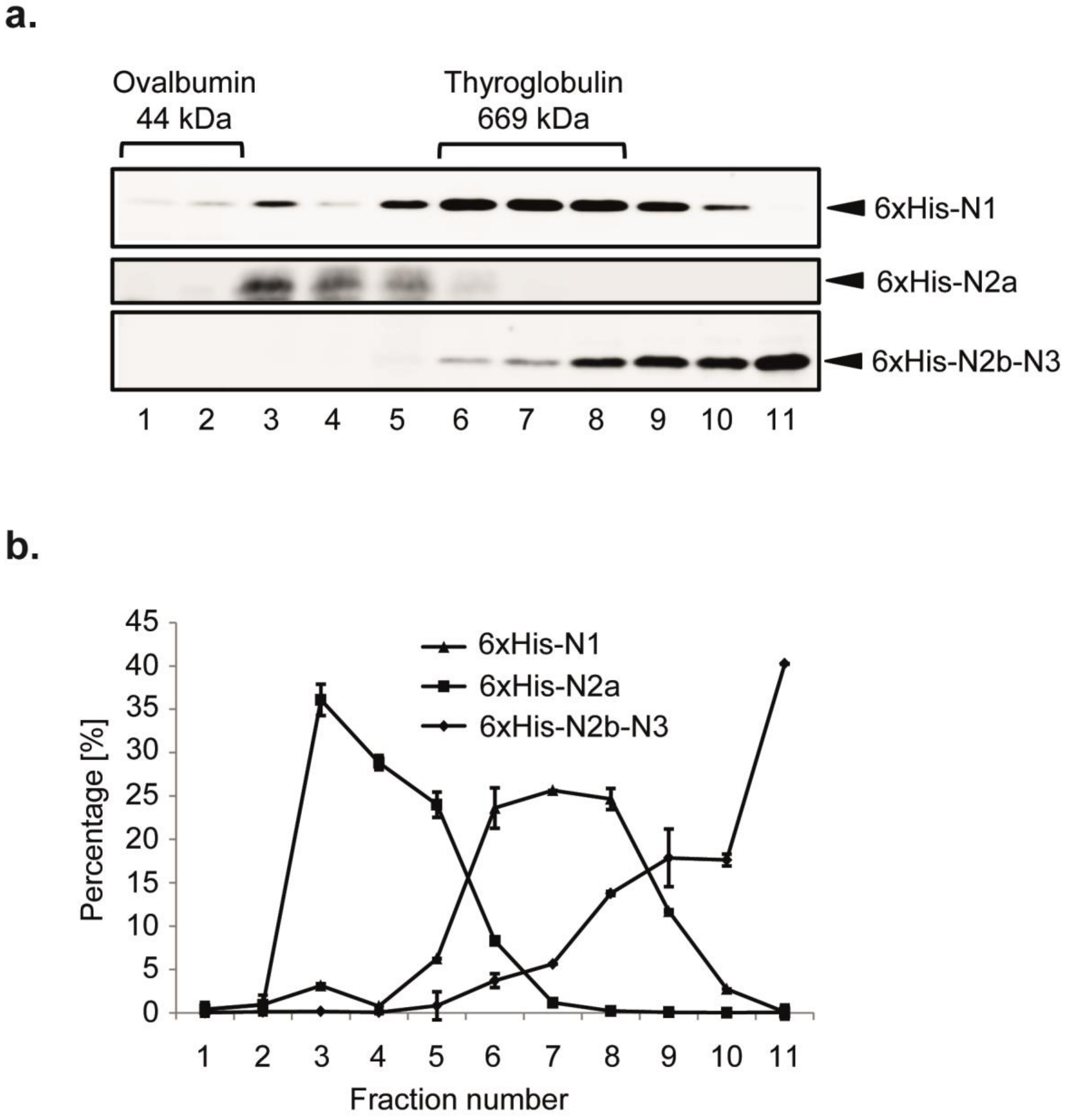

Supplementary Figure S2. Truncated forms of MHV N protein are able to form oligomers. (a) The bacterial extracts from *E. coli* expressing 6xHis-tagged N1, N2a and N2b-N3 fragments were fractioned and analysed as in Fig. 1c. Only part of the western blot images are shown in the figure. (b) Quantification of the immunoblots presented in panel (a) plus SD (n=3).

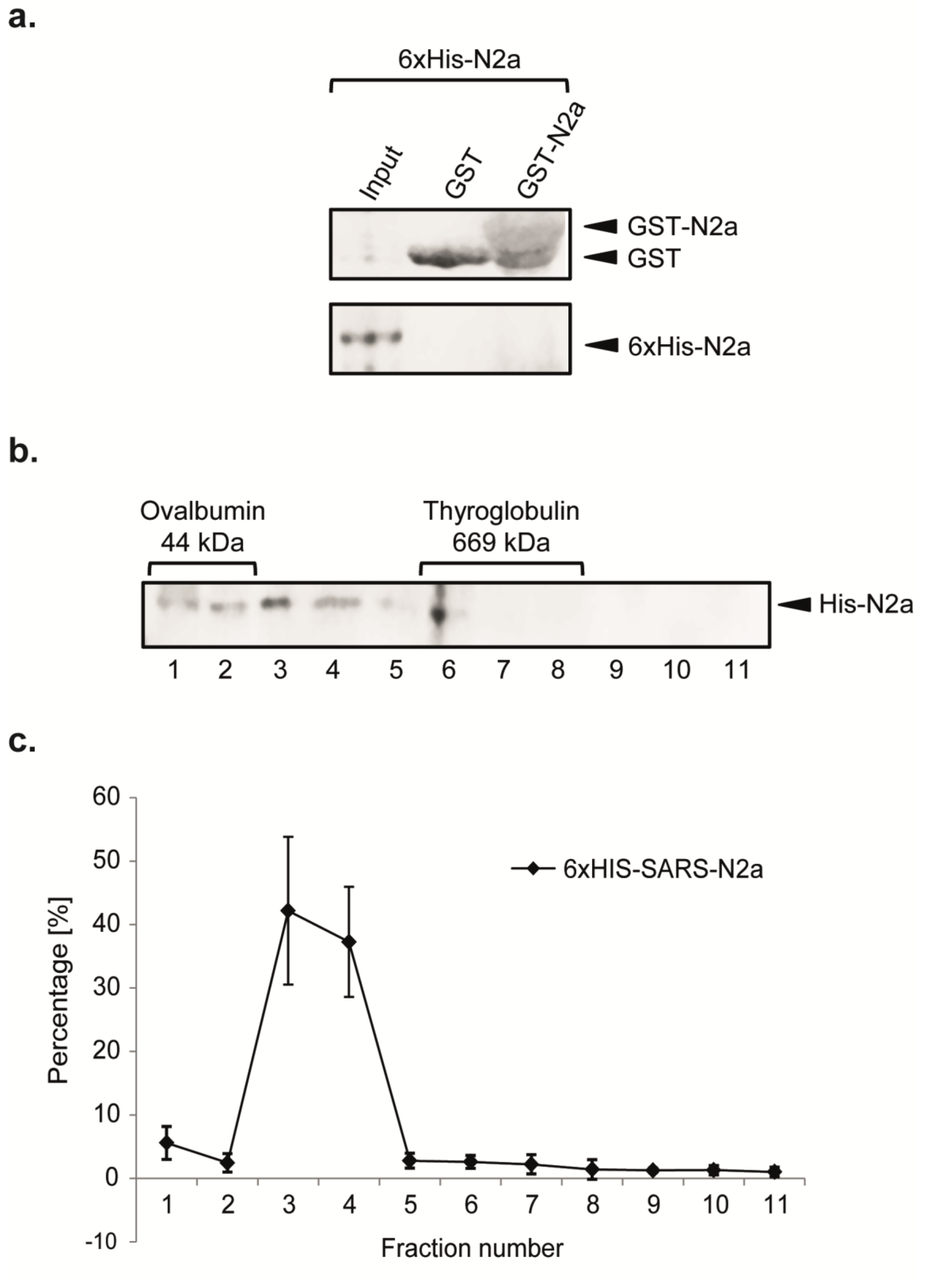

Supplementary Figure S3. The N2a truncated forms of SARS-CoV N protein is unable to self-interact and form oligomers. (a) A bacterial extract from *E. coli* expressing 6xHis-tagged SARS-CoV N2a (input) was incubated with immobilized GST or GST-N2a protein.

Precipitated proteins were eluted in SDS-sample buffer and analyzed by western blot using an anti-His monoclonal antibody. Only part of the western blot images is shown in the figure. The GST and GST-N amount were assessed by staining the PVDF membrane with Ponceau Red. (b) The bacterial extracts from *E. coli* expressing 6xHis-tagged N2a fragments were fractioned and analysed as in Fig. 1c. Only part of the western blot images are shown in the figure. (c) Quantification of the immunoblots presented in panel (b) plus SD (n=3).
